# Network Propagation with Dual Flow for Gene Prioritization

**DOI:** 10.1371/journal.pone.0116505

**Published:** 2015-02-17

**Authors:** Shunyao Wu, Fengjing Shao, Jun Ji, Rencheng Sun, Rizhuang Dong, Yuanke Zhou, Shaojie Xu, Yi Sui, Jianlong Hu

**Affiliations:** 1 College of Automation Engineering, Qingdao University, Qingdao, China; 2 College of Information Engineering, Qingdao University, Qingdao, China; 3 School of Computer Engineering, Qingdao Technological University, Qingdao, China; University of Jaén, SPAIN

## Abstract

Based on the hypothesis that the neighbors of disease genes trend to cause similar diseases, network-based methods for disease prediction have received increasing attention. Taking full advantage of network structure, the performance of global distance measurements is generally superior to local distance measurements. However, some problems exist in the global distance measurements. For example, global distance measurements may mistake non-disease hub proteins that have dense interactions with known disease proteins for potential disease proteins. To find a new method to avoid the aforementioned problem, we analyzed the differences between disease proteins and other proteins by using essential proteins (proteins encoded by essential genes) as references. We find that disease proteins are not well connected with essential proteins in the protein interaction networks. Based on this new finding, we proposed a novel strategy for gene prioritization based on protein interaction networks. We allocated positive flow to disease genes and negative flow to essential genes, and adopted network propagation for gene prioritization. Experimental results on 110 diseases verified the effectiveness and potential of the proposed method.

## Introduction

Disease gene prediction is an important task in bioinformatics. It aims to discover potential disease genes based on known disease genes and omics data, such as metabolic pathways and protein-protein interactions, by utilizing machine learning and complex network theory. It is very important to understand the pathogenesis of hereditary diseases and improve the quality of diagnosis [1].

As a meaningful strategy of disease gene prediction, gene classification aims to construct a binary classification model to automatically determine whether an unknown gene is a disease gene. To effectively distinguish disease genes from non-disease genes, some researchers have utilized sequence-based characteristics to construct classifiers [2]. At the same time, the hypothesis that the neighbors of disease genes are likely to cause diseases prompted scholars to exploit the topological features in protein-protein interaction networks for detecting disease genes [3]. Many studies have explored the integration of various types of features [4–6]. Although gene classification has brought some success, two major problems still exist. First, gene classification selects negative samples (non-disease genes) from unknown genes. However, there are also unrecognized disease genes (false negative samples) that may seriously affect the construction of an accurate classifier [5]. Second, generally, gene classification cannot predict associations between genes and diseases [3, 4, 6]. Only a few disease genes have been verified for each hereditary disease, which is insufficient to train an excellent classifier.

Unlike gene classification, gene prioritization can overcome the two problems mentioned above. The main idea of gene prioritization can be described as follows. Given a disease and its known disease genes, gene prioritization estimates the similarities between unknown genes and known disease genes according to omics data; then, the similarities are sorted in descending order and the top ranked genes are classified as potential disease genes. This provides a convenient method for biomedical experts to select top ranked genes on which to perform experimental verification. The omics data discussed in this paper is protein-protein interaction data. In recent years, gene prioritization based on protein-protein interaction networks has become a hot research topic in bioinformatics [1, 7]. The basic idea is to discover potential disease genes that are closer to or have more interactions with known disease genes.

Gene prioritization can be divided into two types: local distance measurements and global distance measurements. Local distance measurements detect disease proteins according to the local interaction network structure, such as counting the number of known disease proteins in the direct neighbors (Direct Neighbors [8, 9]), or computing the average shortest path to known disease proteins (Shortest Path [10, 11]). Local distance measurements are simple and have low computational complexity, but their performance has been shown to be unsatisfactory. Thus, global distance measurements that can take full advantage of global topological structure have received increasing attention. Random walk with restart [7, 12], kernel diffusion [7] and network propagation [13] are classical global distance measurements. They can effectively detect potential disease genes, which have a high number of interactions with known disease genes. A detailed introduction about gene prioritization has been previously published [14, 15].

One limitation is that global distance measurements may mistake hub proteins with high betweenness for potential disease genes, while hub proteins are probably essential proteins. Thus, it is necessary to identify a method to further determine if the hub proteins are essential proteins, disease proteins or other proteins.

The existing research on protein interaction network analysis is mainly focused on differences in topological importance between essential proteins, disease proteins and other proteins (unknown proteins) [16, 17]. So far, few studies have exploited essential proteins to distinguish disease proteins from other proteins, except our recent research. Our recent study showed that, compared with other proteins, disease proteins are topologically more important [18]. And, disease proteins are closer to the center of the protein interaction network, but are not well connected with essential proteins. We propose that if there are too many essential proteins as neighbors of a candidate protein, the protein is unlikely to cause diseases. However, our recent study only analyzed the proportions of essential proteins among 1-direct neighbors (nearest neighbors) and 2-indirect neighbors (1-direct neighbors’ nearest neighbor [3]) of disease proteins [18]. Thus, more evidence is required to support this new hypothesis.

This paper systematically analyzed the topology associations between disease proteins and essential proteins within protein interaction networks. Empirical results demonstrated that disease genes are not well connected with essential genes. Furthermore, we improved the network propagation method according to the new hypothesis. The main idea is similar to two competing pathogens spreading on a network [19]. We assume that known disease proteins carry positive flow, while essential proteins carry negative flow. And network propagation is considered as the competition between disease proteins and essential proteins. Proteins with more positive flow trend to cause diseases, while proteins with more negative flow are probably non-disease proteins. Thus, by network propagation we can find potential disease proteins that have more interactions with known disease proteins (indicating that they probably have similar functions), but fewer interactions with essential proteins (suggesting that the disease proteins are not well connected with essential proteins). Experimental results on 110 hereditary diseases verified the effectiveness and potential of the proposed method.

## Materials and Methods

### Human gene list, hereditary disease list and human protein-protein interaction data

The disease gene list was downloaded from the Online Mendelian Inheritance in Man database (OMIM) [20]. We selected 2931 disease genes with tag “3” from 6285 entries. Genes with tag “3” have been verified by the presence of a mutation. Then, we obtained housekeeping genes from the research of Chang et al. [21]. Housekeeping genes are universally expressed in normal tissues or cells and are vital to maintaining fundamental life activities. Thus, housekeeping genes can be deemed as essential genes [16].

We obatined 110 hereditary diseases and corresponding disease genes from Kohler et al. (http://download.cell.com/AJHG/mmcs/journals/0002-9297/PIIS0002929708001729.mmc1.zip). Kohler et al. [7] collected the associations between genetic diseases and disease genes from OMIM, domain knowledge and medicinal literatures. Here, 110 diseases are accounted for by 794 disease genes; there were 681 unique genes listed (one gene may cause more than one disease).

The human protein interactions were downloaded from the i2d (http://ophid.utoronto.ca/ophidv2.204/) and STRING (http://string-db.org/) databases. Table 1 lists the statistics of networks constructed based on the protein interactions. The i2d database uses proteins as interactors. Thus, we mapped genes to proteins according to the UniProt database (http://www.UniProt.org). Unlike the i2d database, the STRING database uses genes as interactors, and provides a score to evaluate the reliability between two interactors. Similar to Kohler et al. [7], we set a threshold score of 0.4 to extract unweighted interactions. We integrated all the data from the two databases to construct a larger network (this paper refers it to “integrated protein interaction network”) for disease gene prediction.

**Table 1:** Networks used in this work.

Network	Number of interactors	Number of interactions	Number of interactors in the largest component	Number of interactions in the largest component
i2d	14060	117002	13980	116956
STRING	11632	128104	11502	128017
All data source	15215	200044	15106	200012

In this paper, we annotated essential proteins/genes and disease proteins/genes as *E* and *D* respectively, and the remaining proteins/genes (*O* = ¬(*E* ⋃ *D*)) were treated as other proteins/genes. Table 2 and Table 3 list the statistics of different types of interactors in the protein interaction networks constructed based on the i2d and STRING databases. For the sake of brevity, ¬*D* ⋂ *E* is denoted by *E* ^−^ and ¬*E* ⋂ *D* is denoted by *D* ^−^.

**Table 2:** Statistics of the proteins in the protein interaction network constructed based on the i2d database.

	Number of proteins	Number of proteins in the largest component
*D*	2490	2481
*E*	1942	1938
*E* ∩ *D*	297	297
*D* ^−^	2193	2184
*E* ^−^	1645	1641
*O*	9925	9858

**Table 3:** Statistics of the genes in the protein interaction network constructed based on the STRING database.

	Number of genes	Number of genes in the largest component
*D*	2339	2310
*E*	1706	1696
*E* ∩ *D*	277	275
*D* ^−^	2062	2035
*E* ^−^	1429	1421
*O*	7864	7771

### Analysis of the topology associations between disease proteins and essential proteins

Essential genes were initially considered to be stable genes unaffected by other factors. However, recent studies have indicated that the expression of essential genes can be influenced by other factors, such as diseases [22–24]. Our recent study analyzed the associations between disease genes and essential genes in the protein interaction network. Empirical results demonstrated that even though non-essential disease proteins are closer to essential proteins, the proportions of non-disease essential proteins among 1-direct neighbors of non-essential disease proteins are similar to those of other proteins, and the proportions of non-disease essential proteins among 2-indirect neighbors of non-essential disease proteins are statistically smaller than those of other proteins. This finding illustrates that disease proteins are not well connected with essential proteins. In this paper, we systematically study the topology associations between disease proteins and essential proteins.

*n* neighbors of node *i* are defined as node set $Q_{i}^{n}$, in which the shortest path of each element to node *i* is *n*. Here, *n* is a positive integer. For instance, $Q_{i}^{1}$ is the set of direct neighbors of node *i*. We intend to compare the differences of the proportions of non-disease essential proteins among *n* neighbors of non-essential disease proteins and other proteins. For the sake of brevity, the intersection of set $Q_{i}^{n}$ and set *E* ^−^ is denoted by $Q_{E_{i}^{-}}^{n}$, $Q_{E_{i}^{-}}^{n}=Q_{i}^{n}⋂E^{-}$; the size of set $Q_{i}^{n}$ is denoted by $q_{i}^{n}$, $q_{i}^{n}=|Q_{i}^{n}|$; the size of set $Q_{E_{i}^{-}}^{n}$ is denoted by $q_{E_{i}^{-}}^{n}$, $q_{E_{i}^{-}}^{n}=|Q_{E_{i}^{-}}^{n}|$. In this paper, the proportion of non-disease essential proteins among *n* neighbors of node *i* is defined as follows.

$$p_{E_{i}^{-}}^{n}=\frac{q_{E_{i}^{-}}^{n}}{q_{i}^{n}}$$ (1)

In this paper, $\left\{p_{E_{i}^{-}}^{n}|i\in X\right\}$ is denoted by $P_{E^{-}X}^{n}$ and the median of $P_{E^{-}X}^{n}$ is denoted by $Md(P_{E^{-}X}^{n})$.

### Gene prioritization

In this work, the network propagation method was adopted to detect disease genes.

Network propagation on a network can be understood as simulating a process, in which nodes iteratively pump flow to their neighbors [13]. A node would pump equal flow to each of its direct neighbors for each timestamp. We denote the network as *G* = (*V*, *L*). Here, *V* is the node set of the network and *L* is the edge set of the network. Given one positive unit flow to node *x*, the flow pumped from node *x* to node *y* is *W*(*x*, *y*) = *A*(*x*, *y*)/*k*(*x*). Here, *k*(*x*) is the degree of node *x*, **A** is the adjacency matrix, and **W** denotes the normalized adjacency matrix. *A*(*x*, *y*) = 1 if, and only if, (*x*, *y*) ∈ *L*; otherwise, *A*(*x*, *y*) = 0. In this way, we can evaluate the similarities between other nodes and node *x* based on the network structure.

Furthermore, in order to combine prior knowledge (nodes that are allocated prior information should have more flow) and network structures (adjacent nodes are assigned with similar flow), network propagation can be defined as follows:

$$F^{t+1}=(1-\alpha )WF^{t}+\alpha Y$$ (2)

Here, *F* ^*t*^ is a vector in which *i*-th element holds the flow allocated to node *i* at timestamp *t*, *α* is a parameter controlling the prevalence of prior information *Y* (a ∣*V*∣ * 1 vector), and *F* ^1^ = *Y*. Given *F* ^*t*+1^ = *F* ^*t*^, we can obtain the steady-state solution *F* ^∞^ to equation (2):

$$F^{\infty }=\alpha {\left(I-(1-\alpha )W\right)}^{-1}Y$$ (3)

Denote *α*(**I** − (1 − *α*)**W**)^−1^ as **S**, and the element *S*(*x*, *y*) stands for the similarity between node *x* and *y*. Given a hereditary disease *h* and its known disease genes *T*_*h*_, the similarity of candidate gene *x* with disease genes can be computed as follows.

$$F^{\infty }(x)=\sum_{y\in T_{h}}S(x,y)$$ (4)

The above equation is a particular solution of equation (2) when each disease gene of disease *h* is assigned +1 unit flow for the prior information *Y*. According to the above equation, we can rank the candidate disease genes. This is a global distance measurement for disease gene prediction, called “*NP*_*D*_”. *NP*_*D*_ is mainly based on the well-known hypothesis that the neighbors of disease genes are likely to cause the same or similar diseases. Because *NP*_*D*_ can effectively exploit global topological structures, such as dense indirect interactions between disease proteins, the performance is obviously better than local distance measurements.

We intend to exploit a new hypothesis that, if too many non-disease essential proteins exist as neighbors of a candidate protein, the protein is unlikely to cause diseases. According to this hypothesis, we can assign −1 unit flow to each non-disease essential protein for the prior information *Y*. The dissimilarity of candidate gene *x* with non-disease essential genes can be computed as follows.

$$F^{\infty }(x)=-\sum_{y\in E^{-}}S(x,y)$$ (5)

In this paper, this is termed “*NP*_*E*_”.

This paper integrates the above two hypotheses. We allocate positive flow to the disease proteins and negative flow to the non-disease essential proteins to set the prior information *Y*. Additionally, we ensured that the amount of positive flow is equal to that of negative flow. In the experiment, +1 unit flow was assigned to all disease proteins, while −1 unit flow was allocated to all non-disease essential proteins. The rank of candidate gene *x* was assigned with its score defined as

$$F^{\infty }(x)=\frac{1}{\left|T_{h}\right|}\sum_{y\in T_{h}}S(x,y)-\frac{1}{\left|E^{-}\right|}\sum_{y\in E^{-}}S(x,y)$$ (6)

This paper named the new strategy “*NP*_*D*&*E*_”.

To validate the new strategy, we utilized Leave-One-Out Cross-Validation [7] in the experiments. Given a hereditary disease and the corresponding disease genes (suppose the total number of disease genes is *m*), we selected each disease gene as a test set in turn, while leaving the remaining *m* − 1 disease genes as the training set. Therefore, we performed trials *m* times, and adopted the mean value of the results as the performance of the method. In this paper, we used enrichment-analysis [7] and AUC-analysis [25] to evaluate the performance for detecting disease genes.

Enrichment Score is a typical evaluation index for gene prioritization. For each disease gene used as a test gene, we selected 100 closest genes to the gene on the same chromosome to construct a candidate gene list (including the test gene). If the final flow allocated to the test gene is ranked *r*_*th*_, the Enrichment Score is $\frac{50}{r}$. If the test gene has the same flow as other candidate genes, it is ranked last among them. Additionally, if the protein encoded by the test gene is not in the protein-protein interaction network, we consider the rank to be 100 (Enrichment Score is 0.5). In the experiments, we obtained two results for Enrichment Scores. One is termed “*Enrichment score 1*” and includes disease genes not in the protein-protein interaction network. The other is termed “*Enrichment score 2*” and eliminates disease genes not in the protein-protein interaction network.

AUC (Area Under ROC Curve) evaluates the performance of gene prioritization according to ROC (Receiver-Operating Characteristic). AUC is the area under the ROC curve. ROC analysis can effectively estimate the performance of binary classifiers, and gene prioritization can be deemed as binary classification by setting a rank threshold [25]. Candidate genes above the threshold are considered as positive samples (disease genes), while genes below the threshold are negative samples (non-disease genes). Given a certain threshold, we can evaluate the sensitivity and specificity of the method. Specificity is the proportion of the true disease genes above the threshold among the total prioritizations. Since there were 794 disease genes for the 110 hereditary diseases investigated, the number of prioritizations in the experiments was 794. Specificity is the proportion of genes below the threshold among all of the candidate genes. ROC curve can be drawn by plotting the Specificity versus (1-Specificity) subject to the threshold separating the prediction class. A detailed introduction about the ROC curve can be found in references [7] and [25].

## Results

### Disease genes are not well connected with essential genes

In this paper, we systematically study the topology associations between disease proteins and essential proteins.

**Fig 1:**
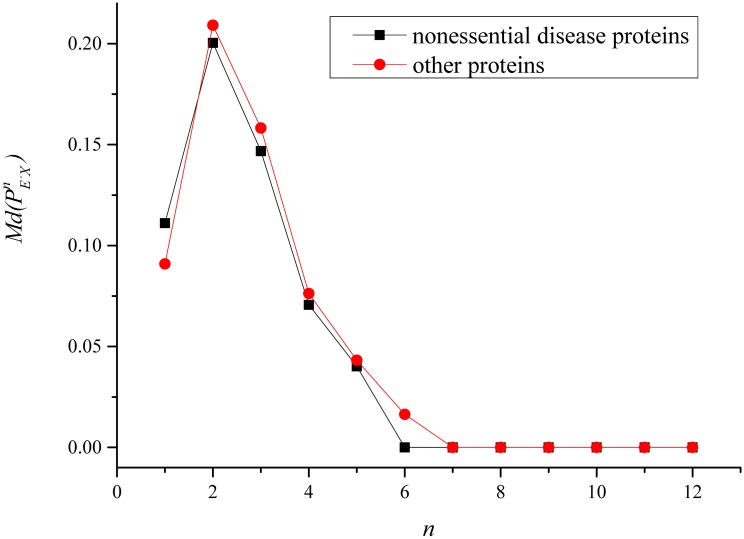
Median values of the proportions of non-disease essential proteins among *n* (*n* ∈ {1, 2, …, 12}) neighbors in the protein interaction network constructed based on the i2d database.

**Fig 2:**
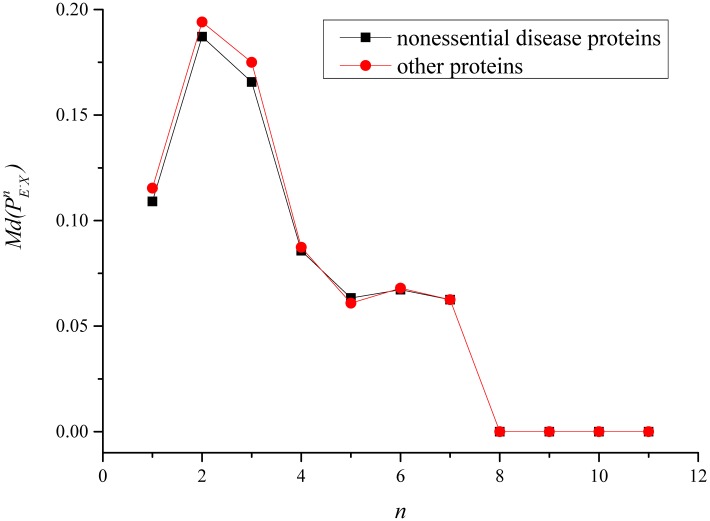
Median values of the proportions of non-disease essential proteins among *n* (*n* ∈ {1, 2, …, 11}) neighbors in the protein interaction network constructed based on the STRING database.

We analyzed the proportions of non-disease essential proteins among *n* neighbors of disease proteins and other proteins, respectively. Fig. 1 and Fig. 2 demonstrate $Md(P_{E^{-}D^{-}}^{n})$ and $Md(P_{E^{-}O}^{n})$ in the protein interaction networks constructed based on the i2d database and STRING databases. As the diameter of the protein interaction network constructed based on the i2d database is 12, *n* ∈ {1, 2, …, 12} in Fig. 1. Similarily, *n* ∈ {1, 2, …, 11} in Fig. 2. The difference between the curves of non-essential disease proteins and other proteins in Fig. 1 and Fig. 2 seems small. However, on the whole, $Md(P_{E^{-}D^{-}}^{n})$ are statically smaller than $Md(P_{E^{-}O}^{n})$ as shown in Table 4 and Table 5. Table 4 and Table 5 provide the statistics of $Md(P_{E^{-}D^{-}}^{n})$ and $Md(P_{E^{-}O}^{n})$ in the protein interaction networks constructed based on the i2d database and STRING databases. The median values of $P_{E^{-}D^{-}}^{n}$ and $P_{E^{-}O}^{n}$ (*n* ∈ {7, 8, 9, 10, 11, 12}) in the protein interaction network constructed based on the i2d database are both 0.00%, and there are no obvious differences. Thus, $P_{E^{-}D^{-}}^{n}$ and $P_{E^{-}O}^{n}$ (*n* ∈ {7, 8, 9, 10, 11, 12}) was ignored in Table 4. Similarily, $P_{E^{-}D^{-}}^{n}$ and $P_{E^{-}O}^{n}$ (*n* ∈ {8, 9, 10, 11}) was ignored in Table 5. Significances between the two protein populations in Table 4 and Table 5 were calculated by the Rank sum test. As shown in Table 4, $Md(P_{E^{-}D^{-}}^{n})$ (*n* ∈ {2, 3, 4, 5, 6}) were significantly smaller than $Md(P_{E^{-}O}^{n})$ in the protein interaction network constructed based on the i2d database. As shown in Table 5, $Md(P_{E^{-}D^{-}}^{n})$ (*n* ∈ {1, 2, 3, 4}) were significantly smaller than $Md(P_{E^{-}O}^{n})$ in the protein interaction network constructed based on the STRING database. Thus, disease genes are not well connected with essential genes in the protein interaction networks.

**Table 4:** Median values of the proportions of non-disease essential proteins among *n* (*n* ∈ {1, 2, 3, 4, 5, 6}) neighbors of nonessential disease proteins (*D* ^−^) and other proteins (*O*) in the protein interaction network constructed based on the i2d database.

	*X* = *D* ^−^	*X* = *O*	p-value
$Md(P_{E^{-}X}^{1})$	11.11%	9.09%	0.5104
$Md(P_{E^{-}X}^{2})$	20.04%	20.92%	7.9795e-06
$Md(P_{E^{-}X}^{3})$	14.68%	15.82%	5.1915e-31
$Md(P_{E^{-}X}^{4})$	7.06%	7.62%	4.9202e-20
$Md(P_{E^{-}X}^{5})$	4.01%	4.32%	1.9152e-09
$Md(P_{E^{-}X}^{6})$	0.00%	1.64%	3.2285e-19

**Table 5:** Median values of the proportions of non-disease essential proteins among *n* (*n* ∈ {1, 2, 3, 4, 5, 6, 7}) neighbors of nonessential disease proteins (*D* ^−^) and other proteins (*O*) in the protein interaction network constructed based on the STRING database.

	*X* = *D* ^−^	*X* = *O*	p-value
$Md(P_{E^{-}X}^{1})$	10.91%	11.54%	5.6717e-03
$Md(P_{E^{-}X}^{2})$	18.72%	19.41%	5.1421e-06
$Md(P_{E^{-}X}^{3})$	16.57%	17.50%	1.4300e-19
$Md(P_{E^{-}X}^{4})$	8.57%	8.73%	2.5534e-05
$Md(P_{E^{-}X}^{5})$	6.33%	6.09%	4.8792e-12
$Md(P_{E^{-}X}^{6})$	6.72%	6.80%	0.6847
$Md(P_{E^{-}X}^{7})$	6.25%	6.25%	0.2228

Goh et al. explained their finding about topology importance of disease genes by using an evolutionary argument [26]. Similarily, our new finding can also be explained using an evolutionary argument. If disease genes have many interactions with essential genes, mutations of disease genes are likely to seriously affect essential genes. This would probably lead to serious disease or even death. Thus, people whose disease genes have more interactions with essential genes were eliminated over the course of evolution. The existing protein-protein interaction network structure can protect the primary normal functions for life.

### Disease genes prediction for 110 diseases

**Fig 3:**
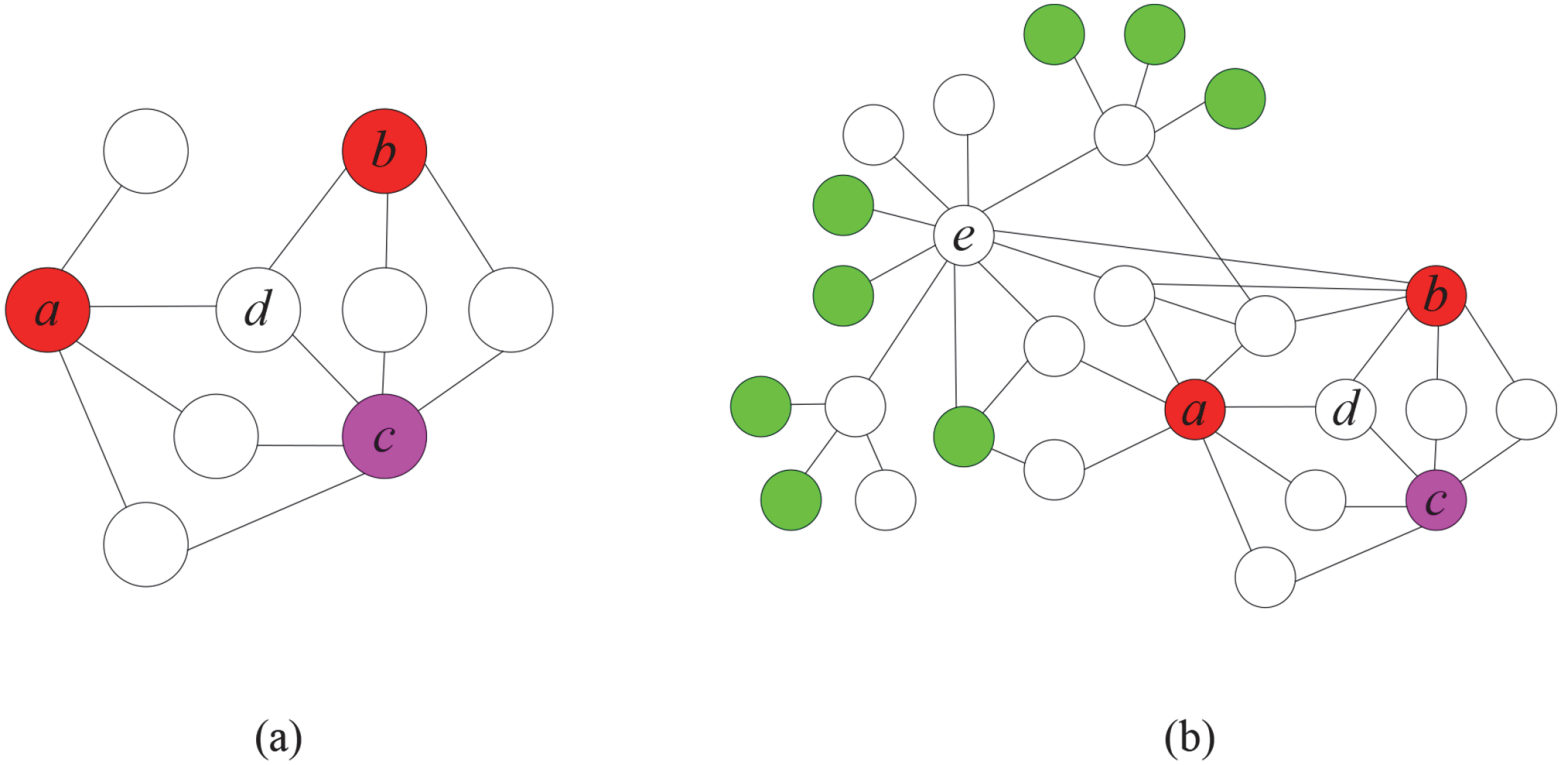
An example of gene prioritization based on network. (a) The disease proteins *a* and *b* are selected as the training set, while *c* as the test disease protein. (b) Global distance measurements may mistake the non-disease hub protein *e* for a disease protein.

Based on the hypothesis that the neighbors of disease genes are likely to cause the same or similar diseases, local distance measurements, such as Direct Neighbors [8, 9] or Shortest Path [10, 11] have been widely used to detect disease genes. However, local distance measurements have many limitations. One major problem is that they cannot effectively detect disease proteins, which are far away from other disease proteins, but have many interactions with them. Thus, Kohler et al. [7] adopted global distance measurements, such as Random Walk with Restart and Kernel Diffusion, to detect disease genes. Global distance measurements can take full advantage of the topological structure of the protein-protein interaction networks, and estimate the similarity between any two proteins based on all of the paths between them. Thus, they can detect candidate disease proteins that have dense interactions with known disease proteins. Fig. 3(a) shows an example. Local distance measurements will mistake the protein *d* for a disease protein, while global distance measurements can correctly identify the disease protein *c*.

Even though the performance of global distance measurements is superior to local distance measurements, hub proteins with high betweenness (essential proteins or other proteins) may be mistaken for candidate disease proteins in some cases. As shown in Fig. 3(b), the non-disease protein *e* has the largest number of interactions with disease proteins and is therefore mistaken for the disease protein. Thus, a novel method is required to select the true disease protein *c*. The empirical analyses in the previous section indicate that disease proteins are not well connected with essential proteins. Additionally, hub proteins with high betweenness that are mistaken for disease genes are probably essential proteins that have numerous interactions with essential proteins. Therefore, we can attempt to avoid mistakes such as those shown in Fig. 3(b) by investigating the proportions of essential proteins among neighbors of candidate proteins. As shown in Fig. 3(b), many essential proteins (green nodes in Fig. 3(b)) exist among neighbors of *e*. This can decrease the probability of mistaking *e* for a disease protein, and enables the correct identification of the disease protein *c*. In the following section, we will demonstrate the advantages of our approach for 110 hereditary diseases.

First, we compared the enrichment score of *NP*_*D*&*E*_, *NP*_*D*_ and *NP*_*E*_ for 110 hereditary diseases with the integrated protein interaction network. As shown in S1 Table, *NP*_*D*&*E*_ can rank all of the disease genes of 18 diseases first (*Enrichment score 2* is 50), such as Alzheimer Disease (4 disease genes), multiple epiphyseal dysplasia AD (5 disease genes) and so on. Specifically, the performance of *NP*_*D*&*E*_ was much better than that of *NP*_*D*_ (the improvement of *Enrichment score 2* was greater than 5) for 41 diseases, and slightly better (the improvement of *Enrichment score 2* was less than 5) for 33 diseases; the performance of *NP*_*D*&*E*_ was the same as *NP*_*D*_ for 20 diseases, and worse than *NP*_*D*_ for 16 diseases.

**Fig 4:**
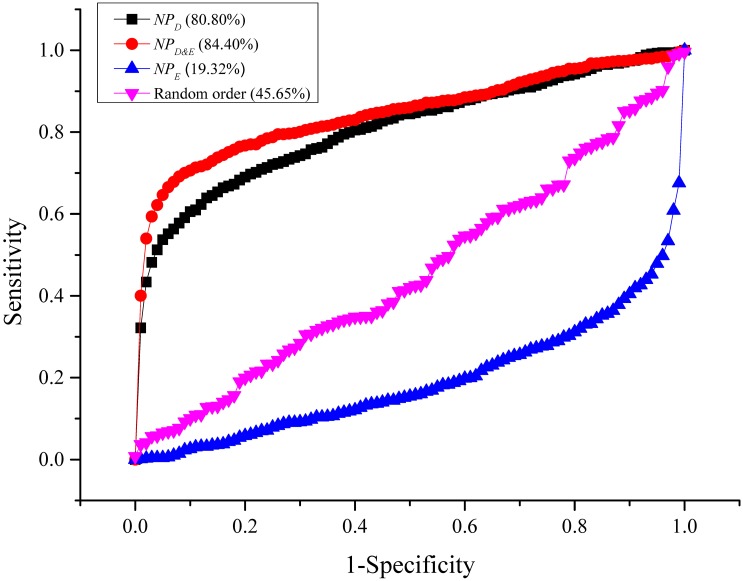
ROC curves.

As shown in Table 6, we performed further statistical analysis on *NP*_*D*&*E*_, *NP*_*D*_ and *NP*_*E*_ for 110 diseases (S1 Table). Compared with *NP*_*D*_, the average of *Enrichment score 1* and the average of *Enrichment score 2* of *NP*_*D*&*E*_ improved by 3.340 and 3.915, respectively. Table 7 presents the probability associated with a one-tailed student’s *t*-test and demonstrates that the improvement in *NP*_*D*&*E*_ is statistically significant. Moreover, we compared the performance of *NP*_*D*_ and *NP*_*D*&*E*_ on monogenic disease, complex disease and cancer, which were divided by Kohler et al. [7]. As shown in Table 6 and Table 7, the improvement in *NP*_*D*&*E*_ for monogenic diseases was the most obvious, and there was a slight improvement in complex diseases. However, the performance of *NP*_*D*&*E*_ in cancer was similar with *NP*_*D*_ (*p* − *value* > 0.99). The reason for this may be that disease genes associated with cancer are usually essential genes, and essential proteins have lots of interactions with other essential proteins, which probably affects the performance of *NP*_*D*&*E*_. Additionally, ROC analysis was adopted to compare the performance of *NP*_*D*&*E*_ and *NP*_*D*_. The disease genes that did not have corresponding proteins in the protein interaction network were excluded in ROC analysis. Fig. 4 indicates that the performance of *NP*_*D*&*E*_ was superior to *NP*_*D*_ with a *t*-test p-value of 3.3307e-016 for *NP*_*D*&*E*_ versus *NP*_*D*_.

**Table 6:** Statistics of the performance (the average values of enrichment score) with disease as a unit.

	*NP*_*E*_		*NP*_*D*_		*NP*_*D*&*E*_	
	*Enrichment score 1*	*Enrichment score 2*	*Enrichment score 1*	*Enrichment score 2*	*Enrichment score 1*	*Enrichment score 2*
Monogenic	0.895	0.941	23.386	26.266	27.181	30.725
Complex	0.829	0.895	10.476	11.029	13.855	14.982
Cancer	0.932	0.951	17.822	18.449	17.855	18.423
All	0.892	0.938	21.370	23.751	24.710	27.666

**Table 7:** One tailed *t*-Tests for Table 6: *NP*_*D*&*E*_ versus Competing Approaches.

*NP*_*D*&*E*_	*NP*_*E*_		*NP*_*D*_	
	*Enrichment score 1*	*Enrichment score 2*	*Enrichment score 1*	*Enrichment score 2*
Monogenic	7.488e-029	3.942e-030	4.484e-010	6.281e-010
Complex	0.007	0.004	0.028	0.018
Cancer	3.034e-004	1.873e-004	0.992	0.994
All	2.585e-032	1.754e-033	4.690e-008	1.270e-008

Next, to compare the ability of *NP*_*D*&*E*_ and *NP*_*D*_ to detect new disease genes, we used the disease genes verified before 2008 as the training set and the disease genes verified after 2008 were used as the test set. The test set consists of 447 new disease genes of 83 diseases verified after 2008 from the OMIM database. Table 8 shows the statistical analyses of the performance of the ability of the two strategies to detect disease genes verified after 2008. *NP*_*D*&*E*_ was able to identify new disease genes more effectively than *NP*_*D*_. According to the statistical analyses, the average rank of disease genes according to the *Enrichment score 2* of *NP*_*D*&*E*_ was $\frac{50}{14.548}\approx 3$. This result implies that *NP*_*D*&*E*_ can assist biomedicine experts to efficiently discover new disease gene with a small amount of medical experiments.

**Table 8:** Statistics of the performance (the average values of enrichment score) with disease as a unit to detect disease genes of 83 diseases verified after 2008. Significances (p-value) between the results of *NP*_*D*_ and *NP*_*D*&*E*_ were calculated by the one tailed student’s *t*-test.

	*NP*_*D*_		*NP*_*D*&*E*_		p-value	
	*Enrichment score 1*	*Enrichment score 2*	*Enrichment score 1*	*Enrichment score 2*	*Enrichment score 1*	*Enrichment score 2*
Monogenic	9.915	11.629	13.579	15.603	7.137e-006	1.006e-005
Complex	6.896	8.478	8.611	12.114	0.064	0.031
Cancer	6.132	7.669	8.484	10.336	0.045	0.041
All	9.047	10.773	12.432	14.548	2.179e-007	1.760e-007

**Fig 5:**
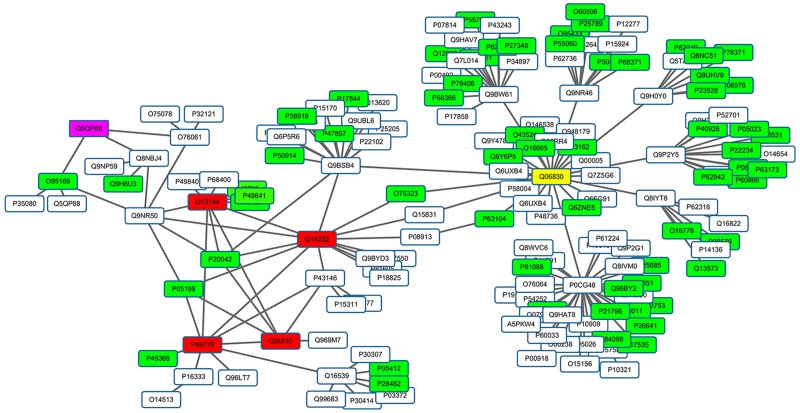
Leukoencephalopathy with Vanishing White Matter Protein-Protein Interaction Network.

Finally, we provided a true example of effectively detecting disease genes by *NP*_*D*&*E*_. Fig. 5 offers the disease proteins of Leukoencephalopathy with vanishing white matter and their interactions in the protein interaction network constructed based on the i2d database. *NP*_*D*&*E*_ was able to correctly identify each disease protein, while *NP*_*D*_ failed to identify the disease protein Q5QP88. In Fig. 5, white nodes stand for other proteins, blue nodes denote non-disease essential proteins, red nodes indicate disease proteins that were correctly identified by *NP*_*D*_, the purple node signifies a disease protein that was not correctly identified (Q5QP88 ranked 14th) by *NP*_*D*_, and the yellow node is a non-disease protein that was mistaken for a disease protein by *NP*_*D*_. Because disease proteins Q13144, Q14232, Q9UI10 and P49770 are closer to each other and have many interactions between them, they can be correctly identified by *NP*_*D*_. However, Q5QP88 is located at a distance from other disease proteins and there are fewer interactions between them. Thus, in the prioritization of *NP*_*D*_, the final flow allocated to Q5QP88 was 1.15e-04 while that for Q06830 was 3.49e-04, and Q06830 was mistaken for the disease protein. The proportion of essential proteins among the neighbors of Q06830 was very high indicating that Q06830 was not a disease protein according to our hypothesis. In contrast to *NP*_*D*_, in the prioritization of *NP*_*D*&*E*_, the flow allocated to Q5QP88 was 9.668e-05 (Q5QP88 ranked 1st) while Q06830 was −8.997e-05 (Q5QP88 ranked last).

## Discussion

Molecular networks describe interactions among molecules that can reflect functional linkages. Thus, network-based methods have been widely researched to discover potential disease genes with similar functions to known disease genes. By taking full advantage of global topology structure, global distance measurements can achieve superior performance compared to local distance measurements. However, some problems exist in the global distance measurements. For example, Yang et al. [27] indicated that network-based methods are limited by detecting potential disease genes only in the small regions of known disease genes. As shown in Fig. 5, global distance measurements may mistake non-disease hub proteins for potential disease proteins. One main cause of the above problems is that the existing network-based methods are designed based on the typical hypothesis that the neighbors of disease genes are likely to cause the same or similar diseases. Thus, the methods can only detect potential disease genes that have high topological similarities with known disease genes.

To solve the above problems, this paper attempted to discover new properties of disease genes by analyzing the topology associations between disease proteins and essential proteins in the protein interaction network. Empirical results demonstrate that disease genes are not well connected with essential genes in the protein interaction networks. The new finding can be utilized to explain the conclusion that disease proteins are topologically more important than other proteins [18].

One major hypothesis of molecular network analysis is that “there is a tight relation between network structure and biological function” [28]. Thus, many studies analyzed the properties of disease genes with protein interaction networks [3, 17, 18, 26], and demonstrated that disease proteins are topologically important [3, 17]. However, Goh et al. [26] indicated that a small amount of essential genes exist in the disease genes, and this may affect the correctness of analyses. Goh et al. selected mouse lethal orthologs of human genes as human essential genes and demonstrated the majority of disease proteins are topologically neutral. Nevertheless, a knockout for their mouse orthologs has not been reported for 60% of disease genes [29]. We analyzed the topology importance of disease proteins by utilizing housekeeping genes as essential genes [18]. Empirical results demonstrated that disease proteins are topologically more important than other proteins. However, a new question was raised: because disease proteins are topologically important, would disease genes seriously affect human survival? Our new finding can answer the question to some extent. Because disease genes are not well correlated with essential genes, disease genes would not seriously affect normal activities. Additionally, our finding provides new insights into understanding of the pathogenesis of diseases.

Based on the new finding, we proposed a new hypothesis that if too many non-disease essential proteins exist as neighbors of a candidate protein, then the protein is unlikely to cause diseases. We proposed a network propagation method based on the typical hypothesis and the new hypothesis. The method not only considers the topological similarities of candidate proteins with known disease proteins but also exploits the topological dissimilarities of candidate proteins with essential proteins. To some extent the method can avoid mistaking non-disease hub proteins as potential disease proteins. Our strategy will be beneficial creating new ideas and new visions for disease gene prediction and will be insightful and helpful for predicting genotype-phenotype associations with the phenome-interactome network [27].

Our future works will be the further studies of the dual flows integration for detecting disease genes based on game theory. Additionally, we intend to apply our strategy to assist molecular diagnosis, in order to speed up the identification of disease genes in next-generation sequencing data [30]. Itan et al. utilized a local distance measurement that adopts shortest path to the core gene for monogenic disorders [30]. It could be beneficial to utilize our new global measurement for improving the quality of molecular diagnosis.

## Supporting Information

S1 Table Enrichment results with the integrated protein interaction network. (DOC)
