# Supplementary material for: Network Propagation with Dual Flow for Gene Prioritization
Source: PLoS One. 2015 Feb 17;10(2):e0116505. doi: 10.1371/journal.pone.0116505 (PMC4331530; doi:10.1371/journal.pone.0116505)
Supplement: S1 Table — (DOC) [file pone.0116505.s001.doc]

**Table S1:** Enrichment results with the integrated protein interaction network.

| **Disease name** | ***NP_E_*** | | ***NP_D_*** | | ***NP_D&E_*** | |
| --- | --- | --- | --- | --- | --- | --- |
|  | ***Enrichment score 1*** | ***Enrichment score 2*** | ***Enrichment score 1*** | ***Enrichment score 2*** | ***Enrichment score 1*** | ***Enrichment score 2*** |
| Achromatopsia | 1.565 | 2.098 | 1.469 | 1.954 | 7.237 | 10.606 |
| Alzheimer Disease | 0.501 | 0.501 | 43.750 | 43.750 | 50.000 | 50.000 |
| Adrenoleukodystrophy | 0.502 | 0.503 | 35.100 | 43.750 | 35.100 | 43.750 |
| Aicardi-Goutieres syndrome | 0.505 | 0.507 | 28.250 | 37.500 | 37.625 | 50.000 |
| Amyloidosis VI | 0.608 | 0.608 | 18.969 | 18.969 | 35.714 | 35.714 |
| Amyloidosis familial visceral | 0.525 | 0.525 | 34.524 | 34.524 | 35.185 | 35.185 |
| Amyotrophic lateral sclerosis | 0.728 | 0.728 | 4.968 | 4.968 | 5.339 | 5.339 |
| Age-Related Macular Degeneration | 1.155 | 1.286 | 4.530 | 5.336 | 7.527 | 8.933 |
| Arthrogryposis | 0.503 | 0.503 | 37.500 | 37.500 | 37.500 | 37.500 |
| Arrhythmogenic right ventricular dysplasisa | 0.920 | 0.920 | 10.690 | 10.690 | 15.429 | 15.429 |
| Atypical mycobacteriosis familial | 0.502 | 0.502 | 41.667 | 41.667 | 37.584 | 37.584 |
| Bardet-Biedl Syndrome | 0.623 | 0.645 | 29.935 | 35.287 | 36.717 | 43.301 |
| Bare lymphocyte syndrome type I | 0.512 | 0.512 | 16.667 | 16.667 | 16.667 | 16.667 |
| Bare lymphocyte syndrome type II | 0.500 | 0.500 | 37.625 | 50.000 | 37.625 | 50.000 |
| Bile-acid synthesis defect congenital | 0.500 | 0.500 | 0.500 | 0.500 | 6.625 | 25.000 |
| Bladder Cancer | 0.525 | 0.525 | 22.768 | 22.768 | 25.282 | 25.282 |
| Brachydactyly | 0.864 | 0.864 | 11.340 | 11.340 | 21.915 | 21.915 |
| Breast cancer familial | 0.935 | 0.935 | 17.985 | 17.985 | 26.022 | 26.022 |
| Cataract autosomal dominant | 0.795 | 0.861 | 19.910 | 24.224 | 23.197 | 28.241 |
| Cerebrooculofacioskeletal syndrome | 0.667 | 0.667 | 31.466 | 31.466 | 33.333 | 33.333 |
| Charcot Marie Tooth Disease | 0.715 | 0.737 | 10.948 | 12.048 | 17.543 | 19.337 |
| Cholestasis | 0.505 | 0.508 | 22.389 | 33.333 | 25.167 | 37.500 |
| Chondrodysplasia punctata | 0.834 | 0.925 | 20.195 | 25.567 | 23.590 | 29.888 |
| Combined oxidative phosphorylation deficiency | 0.542 | 0.542 | 17.444 | 17.444 | 35.417 | 35.417 |
| Congenital central hypoventilation syndrome | 1.554 | 1.554 | 13.508 | 13.508 | 22.917 | 22.917 |
| Congenital myasthenic syndromes | 0.504 | 0.504 | 40.000 | 40.000 | 40.000 | 40.000 |
| Cornelia de Lange syndrome | 0.505 | 0.508 | 21.000 | 31.250 | 33.500 | 50.000 |
| Cutis laxa | 0.500 | 0.500 | 37.625 | 50.000 | 37.625 | 50.000 |
| Dilated cardiomyopathy | 1.114 | 1.146 | 14.124 | 14.841 | 19.122 | 20.102 |
| Distal hereditary motor neuronopathy | 0.859 | 0.919 | 7.115 | 8.217 | 11.198 | 12.981 |
| Ectodermal dysplasia | 0.500 | 0.500 | 50.000 | 50.000 | 50.000 | 50.000 |
| Ehlers Danlos syndrome | 0.737 | 0.767 | 17.105 | 19.180 | 18.780 | 21.065 |
| Elliptocytosis | 0.514 | 0.514 | 32.500 | 32.500 | 43.750 | 43.750 |
| Epidermolysis bullosa | 0.982 | 1.026 | 31.930 | 34.787 | 35.494 | 38.675 |
| Esophageal carcinoma | 0.808 | 0.842 | 12.663 | 14.014 | 6.134 | 6.763 |
| Essential hypertension | 0.662 | 0.676 | 5.526 | 5.983 | 10.662 | 11.585 |
| Familial exudative vitreoretinopathy | 1.486 | 1.979 | 1.156 | 1.485 | 5.848 | 8.523 |
| Familial hyperinsulinemic hypoglycemia | 0.723 | 0.723 | 10.335 | 10.335 | 12.660 | 12.660 |
| Fanconi anemia | 0.501 | 0.501 | 38.972 | 46.667 | 41.750 | 50.000 |
| Fundus albipunctatus | 0.568 | 0.568 | 33.889 | 33.889 | 41.667 | 41.667 |
| Generalized epilepsy with febrile seizures plus | 0.668 | 0.668 | 2.269 | 2.269 | 14.286 | 14.286 |
| Glioma of brain | 1.008 | 1.092 | 9.723 | 11.261 | 9.331 | 10.802 |
| Graves disease | 0.621 | 0.652 | 3.725 | 4.531 | 5.833 | 7.166 |
| Hemochromatosis | 0.500 | 0.500 | 40.100 | 50.000 | 40.100 | 50.000 |
| Hemophagocytic lymphohistiocytosis | 0.653 | 0.653 | 16.305 | 16.305 | 27.669 | 27.669 |
| Hepataocellular carcinoma | 2.417 | 2.417 | 33.513 | 33.513 | 2.774 | 2.774 |
| Hereditary nonpolyposis colorectal cancer | 0.519 | 0.519 | 29.082 | 29.082 | 32.242 | 32.242 |
| Hermansky-Pudlak syndrome | 0.707 | 0.707 | 23.216 | 23.216 | 29.613 | 29.613 |
| Hirschsprung Disease | 0.508 | 0.510 | 34.458 | 41.250 | 37.583 | 45.000 |
| Holoprosencephaly | 0.570 | 0.584 | 29.435 | 35.222 | 30.638 | 36.667 |
| Hyper-IgM syndrome | 0.696 | 0.696 | 11.009 | 11.009 | 20.180 | 20.180 |
| Hypercholesterolemia familial | 0.500 | 0.500 | 50.000 | 50.000 | 50.000 | 50.000 |
| Hypertrophic cardiomyopathy | 0.770 | 0.838 | 23.836 | 29.670 | 25.415 | 31.644 |
| Hypokalemic periodic paralysis | 0.752 | 0.752 | 2.111 | 2.111 | 8.005 | 8.005 |
| Inflammatory Bowel Disease | 0.968 | 1.123 | 3.881 | 5.009 | 7.726 | 10.135 |
| Joubert syndrome | 0.500 | 0.500 | 50.000 | 50.000 | 50.000 | 50.000 |
| Juvenile myelomonocytic leukemia | 0.591 | 0.591 | 20.776 | 20.776 | 32.333 | 32.333 |
| Juvenile myoclonic epilepsy | 0.589 | 0.611 | 11.747 | 14.559 | 25.100 | 31.250 |
| Kallmann syndrome | 0.500 | 0.500 | 25.250 | 50.000 | 25.250 | 50.000 |
| Kartagener syndrome | 0.500 | 0.500 | 33.500 | 50.000 | 33.500 | 50.000 |
| Keratosis palmoplantaris striata | 0.512 | 0.512 | 25.000 | 25.000 | 30.556 | 30.556 |
| Leber congenital amaurosis | 1.890 | 1.890 | 2.652 | 2.652 | 10.934 | 10.934 |
| Leigh Syndrome | 0.571 | 0.632 | 14.463 | 26.279 | 14.217 | 25.824 |
| Leukoencephalopathy with vanishing white matter | 0.521 | 0.521 | 35.625 | 35.625 | 45.000 | 45.000 |
| Limb-Girdle Muscle Dystrophy | 2.400 | 2.717 | 29.681 | 34.544 | 32.445 | 37.770 |
| Long QT Syndrome | 0.556 | 0.556 | 19.201 | 19.201 | 32.407 | 32.407 |
| Lung cancer | 1.004 | 1.004 | 17.660 | 17.660 | 30.271 | 30.271 |
| Maple-syrup urine disease | 0.500 | 0.500 | 50.000 | 50.000 | 50.000 | 50.000 |
| Medulloblastoma | 0.668 | 0.668 | 19.876 | 19.876 | 18.497 | 18.497 |
| Microphthalmia | 2.555 | 3.142 | 3.662 | 4.565 | 6.412 | 8.101 |
| Mitochondrial complex I deficiency disorders | 0.502 | 0.502 | 40.050 | 44.444 | 40.050 | 44.444 |
| Maturity-Onset Diabetes of the Young | 0.808 | 0.963 | 1.637 | 2.206 | 1.804 | 2.456 |
| Multiple Acyl-CoA Dehydrogenase deficiency | 1.000 | 1.000 | 33.602 | 33.602 | 36.111 | 36.111 |
| Multiple epiphyseal dysplasia AD | 0.500 | 0.500 | 50.000 | 50.000 | 50.000 | 50.000 |
| Mycobacterium tuberculosis susceptibility to | 0.772 | 0.826 | 2.834 | 3.300 | 4.101 | 4.821 |
| Myoclonic dystonia | 0.500 | 0.500 | 33.500 | 50.000 | 33.500 | 50.000 |
| Nemaline myopathy | 0.569 | 0.569 | 37.685 | 37.685 | 34.524 | 34.524 |
| Nephronophthisis hereditary | 0.500 | 0.500 | 50.000 | 50.000 | 50.000 | 50.000 |
| Neuronal ceroid lipofuscinosis | 1.758 | 2.261 | 7.993 | 10.990 | 11.464 | 15.850 |
| Night-blindness congenital stationary | 0.525 | 0.535 | 15.007 | 20.810 | 21.810 | 30.333 |
| Non-Insulin-Dependent Diabetes Mellitus | 1.612 | 1.742 | 2.875 | 3.154 | 10.542 | 11.724 |
| Nonbullous congenital ichthyosiform erythroderma | 1.010 | 1.010 | 1.276 | 1.276 | 9.050 | 9.050 |
| Nonsyndromic hearing loss | 0.944 | 1.166 | 6.962 | 10.193 | 15.630 | 23.195 |
| Noonan Syndrome Costello syndrome Cardiofaciocutaneous Syndrome | 0.514 | 0.514 | 27.043 | 27.043 | 43.750 | 43.750 |
| Obesity | 0.666 | 0.679 | 17.319 | 18.720 | 24.289 | 26.272 |
| Osteopetrosis | 10.528 | 10.528 | 20.739 | 20.739 | 20.680 | 20.680 |
| Pancreatic carcinoma | 0.522 | 0.526 | 15.986 | 19.083 | 14.613 | 17.436 |
| Parkinson's disease | 0.521 | 0.524 | 24.028 | 27.389 | 34.438 | 39.286 |
| Prostate cancer | 1.598 | 1.689 | 5.416 | 5.826 | 6.439 | 6.934 |
| Peters anomaly | 1.280 | 1.280 | 1.625 | 1.625 | 6.070 | 6.070 |
| Pheochromocytoma | 0.539 | 0.539 | 34.295 | 34.295 | 25.300 | 25.300 |
| Pituitary dwarfism | 0.600 | 0.600 | 26.056 | 26.056 | 32.386 | 32.386 |
| Polycystic kidney disease | 2.417 | 2.417 | 33.513 | 33.513 | 33.611 | 33.611 |
| Primary microcephaly | 0.576 | 0.576 | 17.754 | 17.754 | 12.837 | 12.837 |
| Primary open-angle glaucoma | 0.607 | 0.607 | 3.778 | 3.778 | 0.611 | 0.611 |
| Progressive external ophthalmoplegia | 0.512 | 0.516 | 26.375 | 35.000 | 25.306 | 33.575 |
| Pseudohypoaldosteronism type I autosomal recessive | 0.503 | 0.503 | 33.333 | 33.333 | 33.333 | 33.333 |
| Pulmonary surfactant metabolism dysfunction | 2.036 | 2.036 | 0.688 | 0.688 | 1.084 | 1.084 |
| Rheumatoid arthiritis | 0.938 | 1.011 | 3.388 | 3.869 | 8.598 | 9.948 |
| Refsum disease | 0.500 | 0.500 | 40.100 | 50.000 | 40.100 | 50.000 |
| Retinitis pigmentosa | 1.068 | 1.210 | 9.119 | 11.274 | 16.930 | 21.037 |
| Severe congenital neutropenia | 0.521 | 0.532 | 5.881 | 8.571 | 0.623 | 0.684 |
| Spastic paraplegia | 1.740 | 2.360 | 1.050 | 1.324 | 1.032 | 1.298 |
| Spinocerebellar Ataxia | 0.548 | 0.558 | 13.488 | 16.086 | 16.966 | 20.259 |
| Spondylocostal dysostosis | 0.500 | 0.500 | 0.500 | 0.500 | 1.444 | 3.333 |
| Stickler syndrome | 0.500 | 0.500 | 50.000 | 50.000 | 50.000 | 50.000 |
| Systemic lupus erythematodes | 0.711 | 0.746 | 1.949 | 2.190 | 9.880 | 11.444 |
| Thyroid carcinoma papillary | 0.590 | 0.603 | 8.412 | 9.542 | 10.315 | 11.718 |
| Waardenburg syndrome | 0.813 | 0.813 | 41.771 | 41.771 | 42.188 | 42.188 |
| Xeroderma pigmentosum | 0.531 | 0.531 | 43.934 | 43.934 | 43.873 | 43.873 |
